# Inpatient healthcare provider bypassing by women and their children in urban Bo, Sierra Leone

**DOI:** 10.11604/pamj.2016.23.146.8706

**Published:** 2016-03-31

**Authors:** Lila C Fleming, Rashid Ansumana, Alfred Bockarie, Joel Alejandre, Umaru Bangura, David Henry Jimmy, Nigel Waters, Heibatollah Baghi, David Stenger, Kathryn H Jacobsen

**Affiliations:** 1 George Mason University, Fairfax, VA, United States of America; 2 Mercy Hospital Research Laboratory, Kulanda Town, Bo, Sierra Leone; 3 United State Naval Research Laboratory, Washington, DC, United States of America

**Keywords:** Healthcare bypassing, choice behavior, hospitalization, urban health Services

## Abstract

**Introduction:**

Bypassing refers to a person's decision to seek care at a healthcare facility that is not the nearest one of its type to the person's home.

**Methods:**

This study examined inpatient care facility bypassing in urban Bo, Sierra Leone using data from 1,980 women with children 15 years of age and younger who were interviewed in 2010-2011. The locations of residential structures and hospitals were identified using a geographic information system (GIS), and the road distances from participating households to the nearest and preferred inpatient care facilities were measured.

**Results:**

Nine inpatient care facilities serve Bo residents, but more than 70% of the participating women reported that the city's main public hospital (Bo Government Hospital), located in the city center, was their preferred inpatient care provider. Participants resided within a median distance of 0.9 km (Interquartile range (IQR): 0.6, 1.8) from their closest inpatient facility, but they would travel a median distance of 2.4 km (IQR: 1.0, 3.3) to reach their preferred providers. About 87% of the women would bypass their nearest inpatient care facility to access care at a preferred provider. Bypassing rates were similar for various demographic and socioeconomic groups, but higher for women living farther from the city center.

**Conclusion:**

Although Bo has a diverse healthcare marketplace, access to affordable advanced care options is limited. Most women in Bo would choose to bypass facilities nearer to their homes to seek the low-cost and comprehensive care offered by Bo Government Hospital.

## Introduction

Healthcare Bypassing refers to a person's choice to seek care from health facilities that are located farther from their homes rather than nearby options [1, 2]. The healthcare facility bypassing literature seeks to understand the reasons behind this choice. The considerations involved in a decision to bypass may include: the perceived severity of illness [2–4]; the number of beds at the healthcare facility nearest to a person's home [1, 5, 6]; the range of services provided at the facility [7, 8]; satisfaction with the care provided by the facility during previous visits [1, 9]; as well as individual characteristics such as age [1, 9], education [1, 5], marital status [1], income [9, 10], and insurance status [3, 4, 10]. Studies examining the factors associated with bypassing have been conducted almost exclusively in high-income countries or in rural areas of low- and middle- income countries (LMICs) [11]. Few studies have focused on bypassing behaviors in urban areas of LMICs. A study from Chad found significant differences in bypassing of primary care services in rural versus urban areas [10]. In urban areas, the search for high quality services was a key factor in bypassing, and individuals with high socioeconomic status were most likely to bypass [10]. A study from Sri Lanka examined the bypassing phenomenon in both rural and urban areas, but only reported results at the aggregate level [2]. Bypassing was common among those who perceived their illness to be severe and were searching for a higher quality of care for their condition [2]. There is a need for more studies on the factors that influence provider selection and bypassing in urban areas of LMICs where there are diverse options for accessing healthcare services from formal and informal providers [12, 13]. The city of Bo, the second largest urban center in Sierra Leone, has a diverse healthcare marketplace that serves an estimated population of about 150,000 people who live in an area of about 30 km^2^ [14]. Government (public) hospitals and clinics in Bo offer care at low or no cost to the patient. Private nonprofit facilities run by religious organizations (missions) and non-governmental organizations (NGOs) may offer affordable care [15]. Private for-profit hospitals are also available but may be expensive. Treatment is also available from traditional healers, herbalists, and pharmacists as well as from nurses and some physicians in private practice who may make house calls (usually as a side business while also being employed by a hospital or clinic). This paper examines bypassing behavior in Bo. The purposes of this study are (1) to determine the preference of inpatient care facility by mothers of children less than 15 years old for themselves and their children in urban Bo, (2) to examine the rate of inpatient care facility bypassing by women for themselves and their children, and (3) to identify the factors that may contribute to a woman's choice to bypass the nearest inpatient healthcare facility from her home for herself or her child when the need for inpatient care arises.

## Methods

A geographic information system (GIS) based representation of the city of Bo was created in ArcGIS (v. 10.1) by the Mercy Hospital Research Laboratory (MHRL) team. The initial data were compiled in 2009 through the use of a participatory GIS (PGIS) approach [14, 16]. Input from municipal authorities and long-term residents of the city allowed for the accurate mapping of geographic features including administrative boundaries, roads and trails, and water bodies. These geographic data are publicly available at OpenStreetMap.org (https://www.openstreetmap.org/#map=14/7.9526/-11.7371) and have been updated over time. For this project, additional features were added to the GIS, including roads, built structures, and healthcare facilities. The PGIS process identified 68 administrative sections (neighborhoods) in Bo. Two sections were selected for a pilot study [15], then 18 of the remaining 66 sections were randomly sampled for inclusion in a citywide health survey. This paper uses data from those 18 sections. Satellite imagery of the rooftops was used to digitize the footprints of all the buildings in the selected 18 sections, then a PGIS approach was used to identify all of the buildings that were used as residences. In total, 1,659 single-family or multi-family residential structures were identified. Because there are few street numbers used in Bo, it is not possible to identify plots based on an address. Instead, an adult representative of each residential building was asked for consent for a member of the research team to use a handheld global positioning system (GPS) unit (with an on average accuracy of <10 meters) to acquire the longitude and latitude (XY) coordinates of the front door of the structure. The field team also collected the XY coordinates of all stationary healthcare providers in Bo. The institutional review boards of Njala University (Bo, Sierra Leone), George Mason University (Fairfax, Virginia, USA), and the U.S. Naval Research Laboratory (Washington, DC, USA) approved the study protocol.

**Figure 1:**
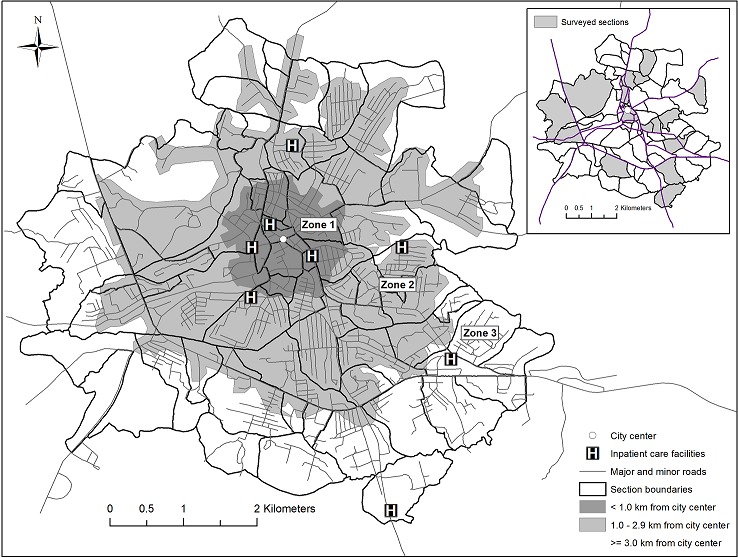
Bo City, Sierra Leone, inpatient healthcare facility locations, road travel distance to a hospital, and sections (neighborhoods) sampled for participation in maternal health surveys

A health survey was conducted in the 18 sections between November 2010 and February 2011 using a two-stage interview process. In the first stage, one consenting adult representative from each household was asked to provide basic information about the demographics and socioeconomic status of the household. Because some residential buildings were home to multiple households, we identified 3,295 households among the 1,659 residences. A consenting adult from 3,286 (99.7%) of these 3,295 households completed the household survey. In the second stage, the 3,975 women age 18 or older who were identified by the household representative as having ever been pregnant were invited to complete a maternal and child health survey. In total, 3,564 (89.7%) of these 3,975 women consented to participate and completed a short interview about their reproductive health, the health of their youngest minor child (if any living children were still less than 15 years of age), and the factors that influenced their provider selection when accessing healthcare for themselves and their children. To ensure the protection of participant data, a trained interviewer recorded all responses on a password protected tablet computer, and at the end of each day the data from the tablets were downloaded to a secure computer and the stored files were removed from the tablet. Two questions were used to examine provider selection for inpatient care. The first question asked *“If you were very sick and needed to be treated for several days and nights away from home, where would you go for care?”* This same question was also asked about each woman's youngest living child. A follow-up question asked which specific healthcare facility or provider a mother would choose for inpatient care. Of the 2,735 (76.7%) participating women who had children 15 years of age or younger, 1,980 (72.4%) named a fixed-location inpatient facility for both herself and her child. Women who named a particular doctor or nurse rather than a fixed-location hospital or clinic were excluded from the analysis, as were women who named a facility that provides only outpatient care. (Facilities named less than 20 times across women and children combined were classified as ones that did not routinely offer inpatient care services and excluded from analysis after local residents confirmed that the facilities were not inpatient care providers.) For this study, a mother or child was classified as having bypassed a healthcare facility if the inpatient healthcare facility nearest in road distance to the home was different from her preferred facility. The Network Analyst tool in ArcGIS was used to identify the nearest inpatient care provider and to calculate the road distance measurements from each woman's home to this facility and to her preferred provider for herself and for her youngest child. This tool was also used to measure the road distance from each residence to the city center, which was defined as the place where three main roads in Bo (Old Gerihun Road, Fenton Road, and Bojon Street) intersect (Figure 1). Geographic data were projected to Universal Transverse Mercator (UTM) coordinate system WGS 84 / UTM Zone 29N. Road segment errors in the geographic data were corrected using a tolerance distance of 3 meters. Residences and facility locations were automatically snapped to the nearest road within 5 meters of the structure.

Four additional questions were used to examine the factors that may influence bypassing: “If cost was not a barrier, would you prefer to go to a different healthcare provider than the one you usually go to when you are sick?” and a three-part paired-comparison question about “When you are choosing where to go for medical care, which is a more important factor?” Using a circular triad method [17], women were presented with three comparisons: cost versus location, cost versus reputation, and location versus reputation. These three questions allowed the highest priority factor to be identified. For example, if a woman chose location, reputation, location in the three paired-comparison questions as her priority, location was her most influential factor. No women provided inconsistent (circular) answers that prevented the top priority from being identified. To assess each participating household's socioeconomic status (SES), an SES index was developed to categorize households into SES tertiles [18]. Factor analysis was used to determine which household-related variables created the best index [19]. Seven household-level variables typically included in an SES index were considered in the factor analysis, and each was categorized on a 3-point scale with 0 indicating low SES, 1 indicating middle SES, and 2 indicating high SES (Table 1) [20, 21]. The five variables with a factor loading value of 0.6 or greater were included in the index [18]. The sum of the 3-point scale values for these five variables (0-10) was used to assign an SES index value. To create SES tertiles, sums of 0 to 4 were classified as low socioeconomic status (SES), scores of 5 and 6 were classified as middle SES, and scores from 7 to 10 were classified as high SES. Based on this method, a total of 656 (33.1%) households were classified as low SES, 766 (38.7%) were classified as middle SES, and 558 (28.2%) were classified as high SES households. Statistical analysis was conducted using SPSS (v. 21) with a significance level of α=0.05. Chi-square tests (x^2^) were used to identify differences in sociodemographic characteristics, SES index, and proximity to the nearest healthcare provider between women who would and would not bypass the nearest inpatient healthcare facility to access the preferred inpatient care provider for herself and her youngest child.

**Table 1:** Factor analysis component loadings

	Score = 2	Score = 1	Score = 0	Component 1 Loadings	Component 2 Loadings	Included in SES index?
*House material*	Concrete block	Mud block	Mud & sticks or other	0.714	0.093	Yes
*Floor material*	Tile	Concrete	Mud or other	0.656	0.161	Yes
*Electricity availability*	Yes	--	No	0.725	-0.207	Yes
*Number of households in the residence*	1	2 or 3	4 or more	-0.095	0.821	Yes
*Number of individuals the in household*	1 to 5	6 to 9	10 or more	-0.068	0.667	Yes
*Toilet type*	Flush latrine	Pit latrine	Bucket	0.480	0.348	No
*Water source*	Piped or bottled	Enclosed pump	Well or spring	0.238	-0.127	No

## Results

Nine inpatient healthcare facilities were identified as preferred providers of inpatient care ( Figure 1). Eight were located within Bo city limits: Bo Government Hospital (BGH), located in the city's center; two government-run community health clinics; four nonprofit facilities (one hospital and three clinics); and one for-profit private hospital. Additionally, the nonprofit Médicins Sans Frontières (MSF) clinic in Gondama, 12 km south of Bo, was listed as a preferred provider for both women and children. (In Bo, the terms “clinic” and “hospital” are not consistently applied to particular types of facilities. Some “clinics” offer inpatient care, and some “hospitals” do not. All nine of the providers listed as preferred offer inpatient care services.) The majority of participants listed BGH, the city's only public referral hospital, as the preferred inpatient provider for themselves (73.9%) and their children (72.8%). Of the remaining women, 18.6% listed a nonprofit, 6.8% listed another government facility, and 0.7% named the for-profit facility as preferred for themselves. For children, these percentages were 20.0% nonprofit, 6.5% public, and 0.8% for-profit. The median distance from the nearest inpatient facility to a home was 0.9 km (Inter-Quartile Range: 0.6, 1.8). The median distance from home to the preferred inpatient care provider was a much longer 2.4 km (IQR: 1.0, 3.3) one-way. Nonprofit providers were the nearest inpatient care providers to more than 60% of homes, but BGH or another public provider was preferred by nearly 80% of the participants. Because of this strong preference for BGH, most participants-87.0% of women and 87.6% of their children-would need to bypass the nearest facility to access care at the preferred facility. Thus, most bypassing for inpatient care in Bo occurs when an individual living near to a nonprofit provider passes by that facility to access care at BGH, which is a median distance of 2.4 km (IQR: 1.1, 3.1) from women's homes. Women who would bypass to reach their preferred facilities for themselves and their children were more likely than non-bypassers to live nearest to a nonprofit provider rather than a public facility, to say that cost was a primary consideration over reputation, to say that they would select a different provider if cost was not a barrier, and to live far from the city center (Table 2 and Table 3). The women most likely to bypass were those who lived farthest from the city center, especially those who lived too far from their nearest inpatient provider to comfortably walk to it. Bypassing rates did not significantly vary by SES or demographic characteristics. Distance from BGH was the primary factor associated with bypassing. Most women who would bypass to access a preferred facility listed BGH as the preferred inpatient care provider. Women preferring BGH were significantly more likely than other women to bypass, and they were also significantly more likely to live far from their nearest providers and to rate cost as a high priority in provider selection (Table 4). Preference for BGH over another facility was not associated with the distance of the home from the city center or a preference for a different provider if cost was not a barrier.

**Table 2:** Inpatient provider selection factors for women (mothers of children less than 15 years old) for themselves, stratified by bypassing behavior, SES index, and the distances from the home to the nearest inpatient provider

		Bypass			SES index			Proximity to nearest provider		
		Yes	No	χ^2^ p-value	High	Low	χ^2^ p-value	≤1.0 km	≥2.0 km	χ^2^ p-value
		n=1723	n=257		n=558	n=656		n=1127	n=444	
		n (%)	n(%)		n (%)	n (%)		n (%)	n (%)	
Nearest provider	Public (Government)	343 (19.9)	193 (75.1)	<0.001*	136 (24.4)	178 (27.1)	0.003*	375 (33.3)	7 (1.6)	<0.001*
	Private nonprofit	1253 (72.7)	57 (22.2)		391 (70.1)	415 (63.3)		643 (57.1)	436 (98.2)	
	Private for-profit	127 (7.4)	7 (2.7)		31 (5.6)	63 (9.6)		109 (9.7)	1 (0.2)	
Preferred provider	Public (Government)	1405 (81.5)	193 (75.1)	<0.001*	451 (80.8)	529 (80.6)	0.595	884 (78.4)	388 (87.4)	0.001*
	Private nonprofit	311 (18.0)	57 (22.2)		101 (18.1)	125 (19.1)		232 (20.6)	54 (12.2)	
	Private for-profit	7 (0.4)	7 (2.7)		6 (1.1)	2 (0.3)		11 (1.0)	2 (0.5)	
Provider selection factor	Reputation	880 (54.9)	161 (69.4)	<0.001*	331 (62.3)	348 (58.7)	0.162	651 (65.1)	265 (63.5)	0.008*
	Cost	718 (44.8)	51 (22.0)		196 (36.9)	234 (39.5)		384 (36.3)	152 (36.5)	
	Location	4 (0.2)	20 (8.6)		4 (0.8)	11 (1.9)		24 (2.3)	0 (0.0)	
Different provider selected if cost not a barrier	Yes	1409 (83.6)	169 (66.3)	<0.001*	456 (82.6)	516 (81.3)	0.547	891 (80.3)	395 (90.4)	<0.001*
	No	276 (16.4)	86 (33.7)		96 (17.4)	119 (18.7)		219 (19.7)	42 (9.6)	
Mother's age (years)	18-24	403 (23.4)	66 (25.7)	0.328	128 (22.9)	154 (23.5)	0.941	278 (24.7)	110 (24.8)	0.164
	25-34	735 (42.7)	97 (37.7)		240 (43.0)	271 (41.3)		475 (42.1)	188 (42.3)	
	≥35	585 (34.0)	94 (36.6)		190 (34.1)	231 (35.2)		374 (33.2)	146 (32.9)	
Age of youngest child (years)	<1	247 (14.3)	47 (18.3)	0.232	80 (14.3)	108 (16.5)	0.691	169 (15.0)	68 (15.3)	0.931
	1-4	844 (49.0)	117 (45.5)		270 (48.4)	311 (47.4)		549 (48.7)	217 (48.9)	
	5-15	632 (36.7)	93 (36.2)		208 (37.3)	237 (36.1)		409 (36.3)	159 (35.8)	
Mother's marital status	Married	1397 (81.5)	214 (84.3)	0.281	454 (81.8)	529 (81.4)	0.925	901 (80.5)	359 (81.0)	0.815
	Not married	318 (18.5)	40 (15.7)		101 (18.2)	121 (18.6)		218 (19.5)	84 (19.0)	
Residence distance from city center (km)	<1.0 km	304 (22.7)	52 (22.9)	<0.001*	133 (29.6)	57 (11.5)	<0.001*	351 (38.4)	0 (0.0)	<0.001*
	1.0-2.9 km	600 (44.7)	144 (63.4)		204 (45.3)	287 (58.0)		409 (44.7)	143 (41.0)	
	≥3 km	437 (32.6)	31 (13.7)		113 (25.1)	151 (30.5)		154 (16.8)	206 (59.0)	
Bypass	Yes	--	--	--	487 (87.3)	564 (86.0)	0.605	930 (82.5)	437 (98.4)	<0.001*
	No	--	--		71 (12.7)	92 (14.0)		197 (17.5)	7 (1.6)	

* Significant at α=0.05

**Table 3:** Inpatient provider selection factors for women (mothers of children less than 15 years old) for their youngest child, stratified by bypassing behavior, SES index, and the distances from the home to the nearest inpatient provider

		Bypass			SES index			Proximity to nearest provider		
		Yes	No	*χ^2^ p-value*	High	Low	χ^2^ p-value	*≤1.0 km*	*≥2.0 km*	*χ^2^ p-value*
		*n=1735*	*n=245*		*n=558*	*n=656*		*n=1127*	*n=444*	
		*n (%)*	*n (%)*		*n (%)*	*n (%)*		*n (%)*	*n (%)*	
*Nearest provider*	*Public (Government)*	347 (20.0)	189 (77.1)	<0.001*	136 (24.4)	178 (27.1)	0.003*	375 (33.3)	7 (1.6)	<0.001*
	*Private nonprofit*	1263 (72.8)	47 (19.2)		391 (70.1)	415 (63.3)		643 (57.1)	436 (98.2)	
	*Private for-profit*	125 (7.2)	9 (3.7)		31 (5.6)	63 (9.6)		109 (9.7)	1 (0.2)	
*Preferred provider*	*Public (Government)*	1382 (79.7)	189 (77.1)	<0.001*	442 (79.2)	519 (79.1)	0.555	870 (77.2)	375 (84.5)	**0.013**
	*Private nonprofit*	347 (20.0)	47 (19.2)		110 (19.7)	135 (20.6)		245 (21.7)	67 (15.1)	
	*Private for-profit*	6 (0.3)	9 (3.7)		6(1.1)	2(0.3)		12(1.1)	2 (0.5)	
*Provider selection factor*	*Reputation*	840 (53.4)	171 (75.6)	<0.001*	317 (60.5)	351 (60.9)	0.392	636 (61.4)	252 (61.8)	0.167
	*Cost*	727 (46.2)	51 (22.6)		207 (39.5)	223 (38.7)		390 (37.7)	156 (38.2)	
	*Location*	5 (0.3)	4 (1.8)		0 (0.0)	2 (0.3)		9 (0.9)	0 (0.0)	
*Different provider selected if cost not a barrier*	*Yes*	1386 (82.5)	174 (73.7)	<0.001*	449 (82.1)	483 (77.2)	0.037*	808 (74.1)	390 (88.8)	<0.001*
	*No*	295 (17.5)	62 (26.3)		98 (17.9)	143 (22.8)		282 (25.9)	49 (11.2)	
*Bypass*	*Yes*	--	--	--	484 (86.7)	569 (86.7)	0.319	942 (83.6)	437 (98.4)	<0.001*
	*No*	--	--		74 (13.3)	87 (13.3)		185 (16.4)	7 (1.6)	

* Significant at α=0.05

**Table 4:** Characteristics of women who prefer Bo Government Hospital (BGH) for themselves and their children

		Women with offspring ≤ 15 years old			Youngest children of participating women		
		BGH*	All other inpatient providers	χ^2^ p-value	BGH*	All other inpatient providers	χ^2^ p-value
		*n=1464*	*n=516*		*n= 1442*	*n=538*	
		*n (%)*	*n (%)*		*n (%)*	*n (%)*	
*Bypass*	*Yes*	1382 (94.4)	341 (66.1)	<0.001**	1359 (94.2)	376 (69.9)	<0.001**
	*No*	82 (5.6)	175 (33.9)		83 (5.8)	162 (30.1)	
*Proximity to nearest provider*	*≤1.0 km*	772 (66.6)	355 (86.2)	<0.001**	764 (67.1)	363 (84.0)	<0.001**
	*≥2.0 km*	387 (33.4)	57 (13.8)		375 (32.9)	69 (16.0)	
*Providerselection factor*	*Reputation*	740 (54.3)	301 (63.8)	<0.001**	694 (53.1)	317 (64.6)	<0.001**
	*Cost*	619 (45.4)	150 (31.8)		311 (46.7)	167 (34.0)	
	*Location*	3 (0.3)	1 21 (4.4)		2 (0.2)	7 (1.4)	
*Different provider selected if cost not a barrier*	*Yes*	1175 (81.8)	403 (80.1)	0.462	1140 (81.4)	420 (81.2)	0.533
	*No*	262 (18.2)	100 (19.9)		260 (18.6)	97 (18.8)	
*Residence distance from city center (km)*	*<1.0 km*	268 (23.6)	88 (20.3)	0.348	268 (23.7)	88 (20.2)	0.328
	*1.0-2.9 km*	529 (46.6)	215 (49.7)		532 (47.0)	212 (48.6)	
	*≥3 km*	338 (29.8)	130 (30.0)		332 (29.3)	136 (31.2)	
*SES Index*	*High*	426 (47.7)	132 (41.2)	0.048**	421 (48.1)	137 (40.5)	0.018**
	*Low*	468 (52.3)	188 (58.8)		455 (51.9)	201 (59.5)	

* Bo Government Hospital.

** Significant at α=0.05

## Discussion

Bypassing inpatient care facilities is a common occurrence in urban Bo. Previous studies have observed that the decision about where to receive health care services is influenced by the facility's quality and reputation and by whether facilities are government or privately run [15, 22]. Most studies suggest that public providers have a poorer reputation than private providers [23–26]. However, in Bo, the main public hospital, BGH, was the strongly preferred option for inpatient care for women and children. The preference for BGH, despite the proximity of many households to other providers, usually private nonprofit facilities, suggests that access to advanced services, such as diagnostic and specialty care, as well as to free or low-cost inpatient care, is a key factor in household-level decision-making about where to access health care. Women from lower SES households may prefer BGH over options nearer to their home because they know the costs of care will be limited. While some private providers offer free or low-cost care, health consumers may not be able to access advanced diagnostic and therapeutic services at those facilities, and they may have to negotiate on prices rather than trusting that prices will be disclosed ahead of time like they are at BGH. Women from higher SES households-the women most likely to say that they would choose a different provider if cost was not a barrier-may consider BGH to be an acceptable and affordable option when compared to the more expensive advanced care options in Freetown, the capital city. However, the paired-comparison questions indicated that reputation was a higher priority for healthcare provider selection than cost or provider location. Thus, the strong preference for BGH expressed by women from both high SES and low SES households may be a sign that, in addition to providing reasonably-priced care, BGH also has a favorable reputation for providing advanced services not available elsewhere in Bo. Although the questionnaire did not directly inquire about the reasons behind a woman's choice of provider for herself or her child, the results may indicate that BGH is providing exceptional care or they may simply reflect the fact that BGH is the only hospital in Bo that offers advanced diagnostics and advanced therapies such as surgery. A lack of competition makes BGH the only viable option for many types of inpatient care services. If other providers in Bo city were offering similar treatment options at a reasonable cost, then BGH's reputation and status as the most preferred provider might quickly dissipate.

Inpatient care requires a particular type of medical provider at a particular type of facility, and for most residents of Bo in 2010-2011, BGH was perceived to be the best option for serious illnesses. This perception may have been related to an innovative healthcare policy implemented nationally in Sierra Leone in April 2010. Under that plan, a variety of free healthcare services were offered to pregnant and breastfeeding women and to children under 5 years of age who sought care at government-run facilities [27]. This initiative may have increased the appeal of BGH for maternal and child health services. Under this new national policy, private healthcare providers may need to examine their cost structures and consider how to appeal to a larger demographic within Bo if they want to maintain and grow their client base. The rising burden of chronic diseases in LMICs is creating new demands for noncommunicable disease diagnosis and long-term management [28, 29]. For example, in Bo there is currently almost no access to anti-hypertensive medications, despite a hypertension rate of about 25% in adults [30]. Both patients and facilities may benefit when new services are offered, such as rehabilitation therapy and other treatments not currently available at BGH. Another option for increasing accessibility and reputation would be for private providers to support transportation to their facilities. There is no public transportation system in Bo, so private (and sometimes costly) arrangements for transportation must be made when a preferred provider is not within easy walking distance of a home. Access to affordable and reliable transportation for severely ill adults and children may enhance a provider's reputation. Healthcare providers can also consider options for alternatively distributed healthcare, such as facility relocation, to increase accessibility [31]. Bypassing practices may vary based on the type of care being sought. A study of primary healthcare facilities in Chad reported a bypassing rate of 54% in an urban area [10]. The rate of bypassing for inpatient care in Bo was much higher, at nearly 90%. Bypassing behavior in Bo might be very different for primary care services, since a greater diversity of public and private formal and informal providers with different price points are available across the city. The decision to bypass in an urban area also appears in other studies to be influenced by an SES status, with higher SES households able to bypass local public facilities to pay for private care [10]. However, in Bo, BGH was a preferred inpatient provider for nearly all demographic and socioeconomic groups, and SES was not a major factor in care seeking. This finding has not been observed by other studies conducted solely in an urban area of an LMIC. Replication studies in urban centers might help to determine whether this is a common finding in places where the inpatient care marketplace is limited. It is important to acknowledge that the questions about the stated preference for healthcare providers were hypothetical ones about plans for future care. In practice, women may not bypass their nearest facility when the need for inpatient care arises even if they would prefer to go elsewhere. And their actual selections of providers might not change even if money was not a barrier. Even with this limitation, the use of road network analysis to examine bypassing in an urban area of an LMIC provides new insights into the factors influencing inpatient bypassing in West Africa.

## Conclusion

In conclusion, a variety of choices of inpatient care providers exist for urban Bo residents, yet the overwhelming preference for BGH, the large government-run facility in the center of the city, indicates a high demand for advanced care services that are at present not being provided at other facilities within the city. This preference may also support the success of the national policy making many types of maternal and child health care free at public hospitals in Sierra Leone. Women in Bo appear willing to bypass nearer facilities and pay for transportation to BGH in order to access inpatient care there. Private facilities, both nonprofit and for-profit, looking to fill a gap in the healthcare marketplace in Bo may want to consider greater transparency about their fees, marketing to improve their reputation and desirability, and offering novel long-term care services, such as chronic disease management and post-surgery rehabilitation.

### What is known about this topic

- Few studies have examined healthcare facility bypassing in urban areas in low-income countries.
- Previous studies suggest public healthcare facilities to be of poorer reputation compared to private facilities.
- Private, more expensive inpatient healthcare facilities, are perceived to be of higher quality and may be less likely to be bypassed by individuals of high socioeconomic status.

### What this study adds

- Inpatient healthcare facility bypassing is a common occurrence in the city of Bo, Sierra Leone.
- Women in Bo were more likely to bypass inpatient nonprofit private facilities to access their preferred healthcare facility.
- A public hospital, Bo Government Hospital, was the preferred inpatient healthcare facility by women of all ages and socioeconomic strata in the city of Bo.
